# Prevalence and Treatment for Alcohol Use Disorders Based on Kentucky Medicaid 2012–2019 Datasets

**Published:** 2022-09-22

**Authors:** Huirong Hu, Riten Mitra, Yuchen Han, Subhadip Pal, Haojiang Huang, Craig J. McClain, Vatsalya Vatsalya, K.B. Kulasekera, Maiying Kong

**Affiliations:** 1 Department of Bioinformatics and Biostatistics, University of Louisville School of Public Health and Information Sciences, Louisville, Kentucky, USA, 40202; 2 Department of Psychiatry and Behavioral Sciences, University of Louisville School of Medicine, Louisville, Kentucky 40202, USA; 3 Department of Pharmacology and Toxicology, University of Louisville School of Medicine, Louisville, Kentucky, USA, 40202; 4 Department of Medicine, Division of Gastroenterology, Hepatology and Nutrition, University of Louisville School of Medicine, Louisville, Kentucky, USA, 40202; 5 Robley Rex Louisville VAMC, Louisville, KY, USA, 40206

**Keywords:** Medicaid, Alcohol use disorder, Prevalence, Medication, Psychosocial therapies

## Abstract

Alcohol use is the leading substance use in the United States. Persons with alcohol use disorder (AUD) face enormous health consequences and family problems. Analysis of Medicaid enrollee data is critical to understand different aspects of AUD and the treatment utilization for patients with AUD. Yearly patient-level data were constructed from the Kentucky 2012–2019 Medicaid claims data. ICD-9-CM and ICD-10-CM codes were used to identify patients with AUD and their comorbid conditions, the 11-digit National Drug Codes were used to identify medication treatments, and procedure codes were used to identify psychosocial and behavioral therapies. Logistic regression models were used to examine factors that were associated with AUD prevalence and AUD treatments. The prevalence of AUD trended up over time. Patients living in metro areas, between ages 45–54, having mental disorders, tobacco use, and with a family history of alcoholism had significantly higher rates of AUD. About 60% of patients diagnosed with AUD had major depressive disorder or anxiety. The treatment utilization for AUD also trended up from 2012 to 2019; however, it was still lower than 25% in 2019. Pharmacological treatments were used in only 2.89% of AUD cases in 2012, which increased to 8.13% in 2019. Psychosocial treatments were used in only 1.59% of AUD cases in 2012 that increased to 18.95% in 2019. The prevalence of AUD trended up over years. However, the treatment utilization for AUD was lower than 25%, even as of 2019. There is an urgent need for comprehensive, evidence-based, personalized AUD treatments.

## INTRODUCTION

The harmful alcohol consumption is a causal factor in more than 200 diseases and injury conditions. Alcohol use becomes the seventh leading risk factor for both disabilities and deaths and contributes to three million deaths each year globally [1,2]. According to the 2019 National Survey on Drug Use and Health (NSDUH) from Substance Abuse and Mental Health Services Administration (SAMHSA), 69.5% of people ages 18 and older report that they drank in the past year in the United States. In this age group, 25.8% of people are in binge drinking (defined as five or more drinks for men and four or more drinks for women on the same occasion at least one day in the past month) and 6.3% are in heavy drinking in the past month (defined binge drinking on the same occasion on each of five or more days in the past 30 days). Nearly 15 million (5.3%) people ages 12 and older are diagnosed with Alcohol Use Disorder (AUD) (the diagnostic domains of alcohol abuse and/or alcohol dependence), including 9 million (6.8%) males and 5.5 million (3.9%) females. An estimated 95000 people died from alcohol-related causes annually in the United States, making alcohol the third-leading causes of death [3]. Excessive alcohol consumption also contributes to enormous health consequences and economic problems [4]. Alcohol dependence is associated with psychiatric conditions, neurologic impairment, liver diseases, and some malignant neoplasms [5–9]. Heavy drinking is associated with various forms of liver disease, such as alcoholic cirrhosis and alcoholic hepatitis. Alcohol misuse cost the United States about $ 249 billion dollars; and it costs Kentucky =−3.2 billion in 2010 [10,11].

The medications approved by the Food and Drug Administration (FDA) to treat AUD include disulfiram, oral naltrexone, extended-release naltrexone, and acamprosate [12–14]. However, only about 6.7% of adults who had AUD in the past year received treatment. It is important to develop effective prevention and treatment strategies that address the physical, behavioral, and social risks resulting from chronic and heavy alcohol drinking, to understand the sociocultural origins of why people respond to alcohol differently. This could also enable to identify the personalized treatments for AUD [15]. The Kentucky Medicaid datasets include the natural history of the diagnoses and treatments for the Medicaid enrollees, and these data could provide a valuable resource for us to examine the causes and effects of AUD, identify potential risk factors of AUD, and seek optimal personalized treatments for patients with AUD.

In this article, we aimed to evaluate AUD in Kentucky Medicaid population, including geographic distribution of AUD prevalence, trends of AUD prevalence and treatments, and to examine the risk factors and comorbid conditions for AUD and its treatments.

## MATERIALS AND METHODS

### Data set and study sample

This study was based on data from the Kentucky Medicaid database from 1/1/2012–12/31/2019 for patients 14 and older. This database included medical claims, containing beneficiary identification (ID) number, demographics and geographic information, the International Classification of Diseases (ICD) 9th edition and 10th edition Clinical Modification (ICD-9-CM and ICD-10-CM) codes, the ICD-9-CM procedure codes, the Healthcare Common Procedure Coding System (HCPCS) procedure codes and the 11-digit National Drug Code (NDC). The yearly segmented data were created as a yearly patient-level dataset by using all the claims, diagnosis codes, procedure codes, drug codes for a patient linked *via* the patient’s unique beneficiary ID number. The yearly patient-level dataset included patients’ demographic information (e.g., age, gender, race, and ethnicity), geographic information (e.g., medical region and metro/non-metro area), the diagnoses of interest (e.g., AUD, mental disorders), and treatment information (e.g., pharmacologic treatment and psychosocial therapies). The University of Louisville Institutional Review Board and the Kentucky Cabinet for Health and Family Services (KCHFS) reviewed and approved the protocol. A data use agreement with the KCHFS Authority permitted access to the Medicaid data.

### Outcome variables

To study the prevalence of AUD, the primary outcome was whether a patient was diagnosed with AUD. A patient was concluded to have AUD if one of the following ICD-9-CM and ICD-10-CM diagnosis codes (Table S1) in the supplement criteria were met: (1) Alcohol abuse (ICD-9 305.0X or ICD-10 F10.1XX); (2) Alcohol dependence (ICD-9 303.XX or ICD-10 F10.2XX); (3) Alcohol associated liver diseases (ICD-9 571.XX or ICD-10 F70.XXX); (4) Alcohol induced mental disorders (ICD-9 291.XX or ICD-10 F10.1XX, F10.2XX); (5) Alcoholic polyneuropathy (ICD-9 357.5 or ICD-10 G26.1); (6) Alcoholic cardiomyopathy (ICD-9 425.5 or ICD-10 I42.6); (7) Alcoholic gastritis (ICD-9 535.3X or ICD-10 K29.2X); and (8) Any mention of alcoholism counseling and alcohol rehabilitation/detoxification [15] identified by ICD-9 procedure codes and HCPCS procedure codes (Table S2) in the supplement [16].

To study the use of both pharmacotherapy and psychosocial treatments for patients with AUD, we identified whether a patient with AUD received any FDA approved drugs (i.e., naltrexone, disulfiram, and acamprosate) and VA approved off-label drug for treating AUD (i.e., topiramate) [17], as identified by the drug codes provided in Table S3 in the Supplement. We also identified whether a patient with AUD received psychosocial and behavioral interventions, which included rehabilitation/detoxification and counseling for AUD. These were identified *via* ICD-9 procedure codes and HCPCS procedure codes (Table S2).

### Covariates

The covariates included the following demographic variables: age in years, gender (male and female), ethnicity (Hispanic and Non-Hispanic), race (White, Black, Other, and missing), and Rural-Urban Continuum (RUC) codes. Age in years was categorized to seven groups (14 to 18, 18 to 24, 25 to 34, 35 to 44, 45 to 54, 55 to 64, 65+). Race and ethnicity were combined to five groups (Non-Hispanic White, Non-Hispanic Black, Non-Hispanic other, Non-Hispanic missing, Hispanic). Areas with a RUC code between 1 to 3 were categorized as metro areas, and areas with a RUC code 4 and above were categorized as a non-metro area. We also constructed a geographical variable that denoted the medical region in which a patient resided. There were 8 medical regions, and these regions covered all 120 counties across the state of Kentucky. The other covariates were tobacco use, family history of alcoholism, and a mental disorder that included anxiety and/or major depressive disorder. There covariates were identified using the diagnosis codes listed in Table S1.

### Statistical methods

We first presented data using descriptive statistics and graphical presentations. From each yearly patient level dataset, we summarized the number of patients who had claims, the number of patients who had AUD, and calculated AUD prevalence rates among the patients who had medical claims. We also summarized the number of patients who had sub-categories of AUD and calculated their percentages among AUD patients (Table 1). To identify risk factors for AUD, we summarized AUD prevalence by gender, age, race and ethnicity, residential geographic information (metro/non-metro), tobacco use, family history of alcoholism, and mental disorders for each year. Here we only presented the results for 2019 in Table 2 under the first block of columns. Logistic regression models were applied to examine the impact of these variables on AUD prevalence, and the odds ratios (OR), 95% Confidence Intervals (CI) and p-values were reported in Table 2 in the second block of columns.

To examine the use of different treatments for patients with AUD and the factors impacting the use of these treatments, we first summarized the number of patients with AUD who received each treatment, which included FDA approved medication, rehabilitation and detoxification, and counseling (Table 3). To identify the factors impacting the treatments for AUD, we summarized treatment usage for AUD by the variables of interests for the year 2019 in Table 4. Logistic regression models were applied to examine the impact of these variables on the use of different treatments for AUD, and the OR, 95% CIs and p-values were reported in Table 5. Statistical significance was set at p-value<0.05. Data management and analysis used R version 3.3.2 software.

## RESULTS

### Results for characterization of Medicaid population and AUD prevalence

We analyzed Kentucky Medicaid data from 1/1/2012 to 12/31/2019 including patients aged 14 or older. The number of patients who had claims had dropped from 471.4 K in 2012 to 464 K in 2013 and rose to 791.6–919.9 K during 2014 to 2017, thereafter slightly dropped to 896.7 K in 2019. Comparing the period 2012–2013 with 2014 to 2019, more patients in the age group 25 to 54 were insured by Medicaid in the latter period, which reflected the impact of the implementation of the Affordable Care Act in 2014 [18]. The percentages insured from 2013 to 2014 were 16.5% to 21% in the 25 to 34 age group, 12.5% to 17.4% in the 35 to 44 age group and 13.4% to 16.0% in the 45 to 55 age group. Among all the patients who had Medicaid claims during 2012 to 2019, 67.7% to 70.9% patients were white, 9.0% to 10.1% patients were black, 15.2% to 18.5% patients did not provide race information, and only 3.2% to 7.0% were Hispanic or other races shown in Table S4.

We first evaluated the prevalence of AUD and its associated risk factors. A patient was determined to have AUD if the patient had diagnosis codes for either alcohol abuse/dependence diagnoses or alcohol associated organ diseases (i.e., alcohol induced mental disorders, alcohol associated liver diseases, alcoholic gastritis, alcoholic polyneuropathy, or alcoholic cardiomyopathy), or the patient had procedure codes for alcoholism behavior and psychosocial treatments. Based on Table 1, the prevalence of AUD was 2.58% in 2012, increased over years and reached 4.21% in year 2019. Among the patients diagnosed with AUD during 2012 to 2019, about 23.95% to 27.73% had alcohol associated organ diseases, about 68.8% to 76.1% had alcohol abuse/dependence but without alcohol associated organ diseases, and about 0% to 3.4% had no AUD diagnosis but had procedure codes for alcoholism behavior and psychosocial treatments. Among patients diagnosed with AUD during 2012 to 2019, about 57% to 62.9% also suffered anxiety and depressive disorders (Table 1).

**Figure 1:**
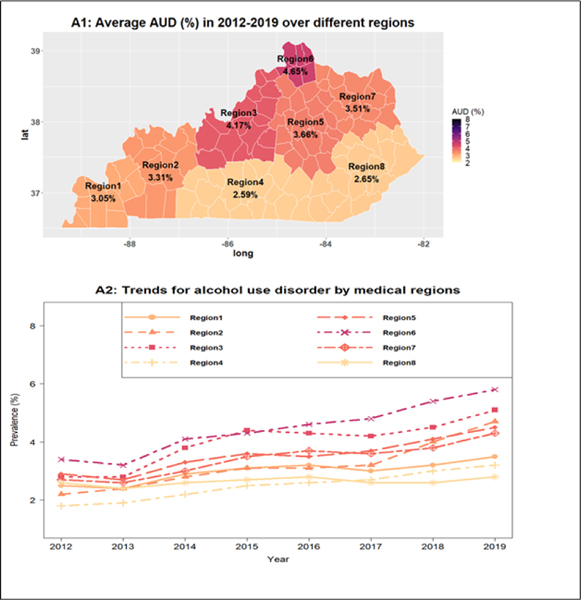
The geographic distribution of the prevalence of AUD. (A1) Average AUD (%) in 2012 to 2019 over different regions. (A2) Trends for alcohol use disorder by medical regions.

**Figure 2:**
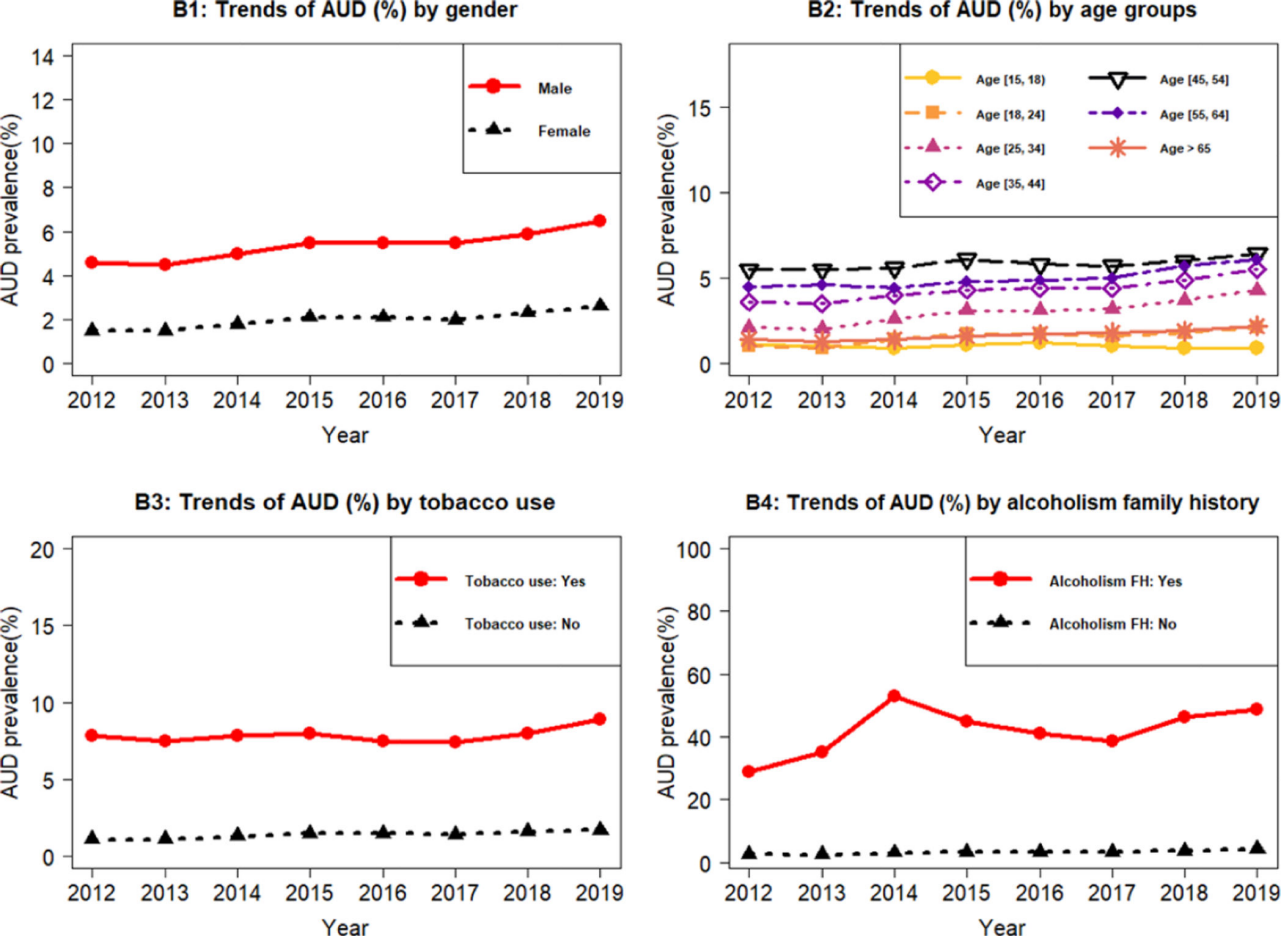
Trends of AUD stratified by gender, age groups, tobacco use, and alcoholism family history. (B1) Trends of AUD (%) by gender. (B2) Trends of AUD (%) by age groups. (B3) Trends of AUD (%) by tobacco use. (B4) Trends of AUD (%) by alcoholism family history.

We examined the association of patients’ variables (e.g., demographics, geographics, tobacco use, family history of alcoholism, mental disorders) with AUD. The average AUD prevalence over different medical regions (calculated as the average of the 8 yearly prevalence rates from 2012 to 2019) are reported in Figure 1 (A1), and their trends are reported in Figure 1 (A2). We found that medical region 3 (Louisville metro and surrounding areas) and medical region 6 (the Northern Kentucky zone close to Cincinnati) had the highest AUD prevalence rates (4.17% to 4.65%), while medical regions 4 and 8 in Southeastern Kentucky had the lowest AUD prevalence rates (2.59% to 2.65%). The prevalence of AUD trended up in almost every medical region (Figure 1 (A2)). Region 6 had the highest prevalence rate, and the annual prevalence rate for this region increased from 3.4% in 2012 to 5.8% in 2019. Medical regions 4 and 8 had the lowest prevalence rates. However, the annual prevalence rates across all regions showed an increasing trend over time (Figure 1 (A2)). We also plotted the AUD trends stratified by patients’ gender (Figure 2 (B1)), age groups (Figure 2 (B2)), and tobacco use (Figure 2 (B3)), and family history of alcoholism (Figure 2 (B4)). We found that males had a significantly higher AUD prevalence rate than females (4.6% versus 1.5% in 2012 and 6.5% vs. 2.6% in 2019) (Figure 2 (B1)). AUD prevalence rates increased as people became older, peaked in the 45–54 age group at about 5% to 6%, and significantly dropped below 2% in the older groups (≥ 65 years old) (Figure 2 (B2)). Patients with tobacco use had higher AUD prevalence (7.8% to 8.9%) than without tobacco use (1% to 2%) (Figure 2 (B3)). Patients with a family history of alcoholism had higher AUD prevalence (29% to 53%) than those without a family history of alcoholism (Figure 2 (B4)).

The descriptive statistics for AUD prevalence stratified by the patients’ variables for year 2019 data are reported in Table 2, and the results based on multiple logistic regression models are also reported in Table 2. Based on Table 2, we found that: (1) non-Hispanic black patients had the highest AUD prevalence among all racial and ethnic groups; (2) patients living in metro areas had higher AUD prevalence (3% to 5.4%) than those living in non-metro areas (2.3% to 3.2%); and (3) Patients with mental disorders had higher AUD prevalence (5% to 7.3%) than those without mental disorders (1.5% to 2.5%). Table 2 and Figure 2 further confirmed that: (1) Males had higher AUD prevalence than females; (2) Patients aged between 45 and 54 years old had the highest AUD prevalence; (3) Patients with tobacco use and family history of alcoholism had much higher AUD prevalence than those without. In particular, the odds for patients with a family history of alcoholism to have AUD were 1.48 times higher than those without, based on 2019 data. Thus, the patients with family history of alcoholism were at a higher risk to suffer from AUD (Table 2).

### Results for characterization of treatments of AUD

**Figure 3:**
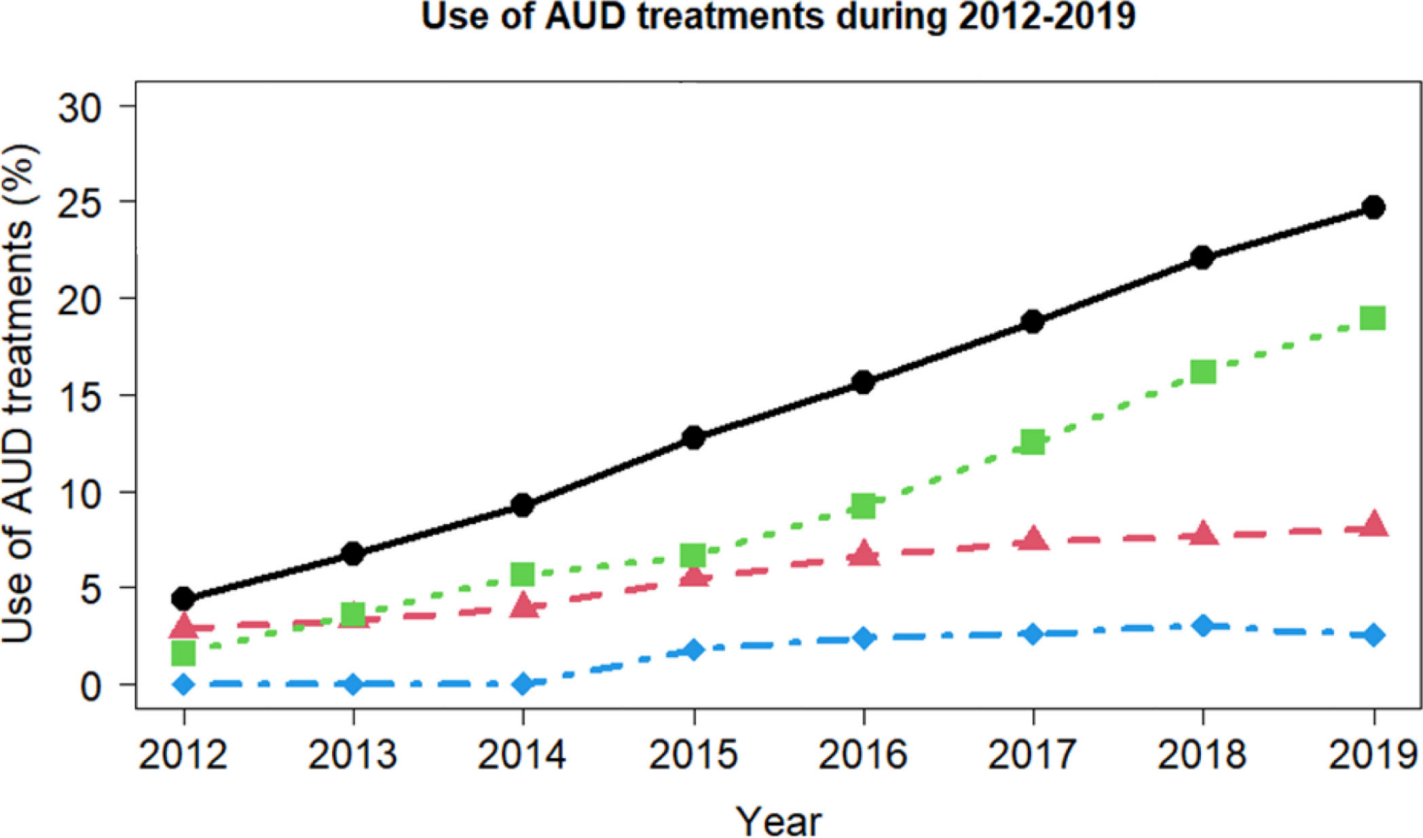
Trends of the use of AUD treatments during 2012–2019 based on Kentucky Medicaid database. Note: (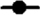) Any treatment, (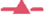) Medication, (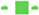) Counseling, (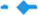) Rehabilitaiton/detoxification.

We observed the utilization of treatments for AUD which included both pharmacological and psychosocial treatments (Table 3 and Figure 3). The pharmacological medications included three FDA approved drugs and one VA approved drug. The utilization of the pharmacological medications had increased from 2.89% in 2012 to 8.13% in 2019. In 2012–2017, the most prescribed medication was topiramate with a usage rate of 2.36% in 2012 increasing to 3.39% in 2017. The prescription of naltrexone increased since 2012, and surpassed topiramate in 2018 with a usage rate of 3.63% and the rate increased to 4.25% in 2019. The utilization of alcohol rehabilitation/detoxification was also low but increased from 0.06% in 2014 to 3.06% in 2018. Alcoholism counseling increased over time from 1.59% in 2012 to 18.95% in 2019. Among the patients with alcoholism counseling, the rate of counseling for at least 3 hours per day and for at least 3 days per week was the highest (Table 3 and Figure 3).

We identified the difference in covariates with utilization of different treatments based on 2019 data. As shown in Table 3, the utilization of any treatment for patients with AUD increased from 4.43% in 2012 to 24.67% in 2019, however, it was still staggeringly below 25%. It would be important to identify factors, which were barriers for patients to receive the needed treatments. For this purpose, we used the 2019 dataset to examine the different treatments with patients’ variables (Table 4).

The descriptive statistics are reported in Table 4, and the results based on multiple logistic regression models are reported in Table 5. We found that:

1. Females were more likely to use medication and counseling than males (10.9% vs. 6.5% for medication; 20.3% vs. 18.1% for counselling; 28% vs. 22.8% for any treatment), but were slightly less use rehabilitation/detoxification (2.4% vs. 2.7%).
2. Patients in the 25 to 34 age group had the highest use of all different treatments (10.9% for medication, 4.7% for rehabilitation/detoxification, 28.7% for counselling, and 35.8% for any treatment), followed by the patients in the 35 to 44 age group (10.9% for medication, 3.4% for rehabilitation/detoxification, 24.4% for counselling, and 31.5% for any treatment). The utilization of treatments decreased as patients became older (Tables 4 and 5). In particular, among patients 65 and older, no patients used medication, only one patient used rehabilitation/detoxification, and 3.3% used counselling.
3. Non-Hispanic Whites had the highest use of treatments in almost every domain of treatments (9.1% for medication, 2.8% for rehabilitation/detoxification, 19.8% for counselling, and 24.4% for any treatment), while the Non-Hispanic Black group had the lowest use of medications (4.3%), and Hispanic patients had the lowest counselling rate (13%) and the lowest rate of any treatment (19.1%).
4. Patients living in non-metro areas were less likely to receive any treatment than those in metro areas (21.6% vs. 26.7% for any treatment; OR 0.962 and 95%CI [0.951, 0.973].
5. Patients with tobacco use were more likely to receive any treatment than those without tobacco use (25.2% vs. 22.5% for any treatment; OR 1.013 and 95%CI [1.003, 1.023]).
6. Patients with a family history of alcoholism were also more likely to receive any treatment than those without a family history of alcoholism (43.6% vs. 24.4% for any treatment; OR 1.148 and 95%CI [1.108, 1.190]).
7. Patients with mental disorders were more likely to receive any treatment than those without mental disorders (29.1% vs. 17.2% for any treatment; OR 1.101 and 95%CI [1.091, 1.110] (Tables 4 and 5).

## DISCUSSION

This study conclusively demonstrated that the prevalence of AUD increased over time from year 2012 to year 2019 and that the utilization of treatment also increased among these patients. Age, gender, ethnicity, medical region, tobacco use, and family history of alcoholism all appeared to be significantly associated with AUD prevalence and treatment for AUD. Among AUD patients, 24% to 32% had other organ diseases such as alcoholic liver diseases; alcohol induced mental diseases, alcoholic polyneuropathy, alcoholic cardiomyopathy, and alcoholic gastritis. These are high cost and life-threatening diseases. Treating patients with AUD in the early stages of the disorder and preventing progression to other organ diseases are particularly important for this vulnerable population.

Although we reported the detailed results on the effect of covariates on AUD based on the 2019 dataset, we also studied these variables based on the 2012 to 2018 datasets and the results were similar (Table S5) in the Supplement. That is, patients in the age group 45 to 54 had the highest AUD prevalence; patients living in metro areas were more likely to have AUD than those living in non-metro areas; and patients with tobacco use, a family history of alcoholism, and/or mental disorders were more vulnerable to suffer from AUD. Therefore, patients with these comorbid conditions need special attention from health providers.

Excessive alcohol use contributes significantly to physical and psychological illness, injury and death, and a wide array of social harm in all age groups. A proven strategy for reducing excessive alcohol consumption levels is to offer a brief conversation-based Rational-Emotive Therapy associated intervention in primary care settings [18,19]. In the Medicaid dataset, a brief conversation-based intervention for alcohol misuse can be captured by the HCPCS code G0443. We found that the number of alcohol misuse counseling increased from 95 cases in 2012 to 607 cases in 2014, and then dropped to 460 cases in 2015. From 2016 to 2019, less than 20 cases per year were recorded for alcohol misuse counseling in each year. This suggests that the brief alcohol misuse counseling should be used for those who have alcohol misuse, which may in turn reduce the harm caused by alcohol drinking and further reduce the severity/prevalence of AUD.

Based on this study, the drug naltrexone had the highest usage in treating patients with AUD. Naltrexone is an opioid receptor antagonist, and it is a common adjunction treatment in opioid addiction. Naltrexone is considered as a safe and effective treatment for patients with alcohol dependence [20]. It reported that in a residential drug and alcohol treatment program, 68% patients had prescription opioids use one month prior to the treatment programs [21]. There were high rates of comorbidity between alcohol and other drug-use disorders; and treatment seeking among persons with alcohol and other drug-use disorders was low [22]. For patients with AUD, the usage of FDA-approved drugs and psychosocial therapies was very low (below 25%) for any treatment. There appear to be treatment barriers, which need to be identified. More off label use of drugs for AUD and other substance use should be considered. There is an urgent need for comprehensive, evidence-based personalized treatments for AUD. The utilization of any treatments was significantly higher for patients living in metro areas than those in non-metro areas. The possible barrier for rural population to get treatment could be the distance to the treating facilities [23,24].

There are some limitations to this study. First, the prevalence rate was calculated based on the patients who had claims in the Medicaid database, which did not account for the patients who enrolled in Medicaid but did not have claims. That is, the denominator in calculating the prevalence rate was not the total Medicaid population regardless of whether a claim was made. However, the claims included any inpatient, outpatient, and emergency visits and any prescriptions. We expect a high percentage (≥ 90%) of patients had claims. Thus, the prevalence of AUD may be slightly overestimated. Nevertheless, the results for the risk factors for AUD prevalence and treatments are valid regardless of this limitation. Second, this study focuses on the Medicaid population rather than general population. That is, the study results represent neither the state nor those insured with private insurance. According to the Kentucky Department for Medicaid Services, less than 40% of the total population in Kentucky was insured by Medicaid before 2013. After expansion, almost 90% of people with low-income were covered by Medicaid [25].

## CONCLUSION

The utilization of pharmacotherapies treating AUD for patients diagnosed with AUD in Kentucky Medicaid population was increased but still lower than 8.2% by 2019. However, among the patients diagnosed with AUD, 60% were diagnosed to have major depression and anxiety and 70% of those were treated with pharmacotherapies for major depression and anxiety. That is, about 42% of patients diagnosed with AUD in Kentucky Medicaid population received pharmacotherapies for major depression and anxiety. In addition, patients with AUD also tended to have high rate of comorbid conditions such as liver diseases. The assessment of treatment utilization for patients with AUD should take account of their comorbid conditions and the pharmacotherapies treating for those comorbid conditions.

## Supplementary Material

Supplement to Manuscript

## Figures and Tables

**Table 1:** The prevalence of alcohol use disorder and their subcategories over time based on Kentucky Medicaid.

Year		2012	2013	2014	2015	2016	2017	2018	2019
Number of patients who had claims		471415	463989	791575	891448	916002	919921	915672	896695
**AUD Specified**	**AUD Patients (n)**	12173	11769	24440	30756	31876	31876	34751	37721
from (1)–(8)	Prevalence (%)	2.58	2.54	3.09	3.45	3.48	3.47	3.8	4.21
(1) Alcohol abuse	Patients (n)	8569	8122	17344	20787	19003	19076	20495	22206
	% in AUD	70.39	69.01	70.97	67.59	59.62	59.84	58.98	58.87
(2) Alcohol dependent	Patients (n)	5309	5222	11611	14708	15367	16742	19368	21414
	% in AUD	43.61	44.37	47.51	47.82	48.21	52.52	55.73	56.77
(3) Alcohol associated liver diseases	Patients (n)	1737	1713	2886	3336	3425	3457	3701	3852
	% in AUD	14.27	14.56	11.81	10.85	10.74	10.85	10.65	10.21
(4) Alcohol induced mental disorders	Patients (n)	1332	1369	3367	5110	5488	4621	5570	5764
	% in AUD	10.94	11.63	13.78	16.61	17.22	14.5	16.03	15.28
(5) Alcoholic polyneuropathy	Patients (n)	61	61	137	163	181	222	273	246
	% in AUD	0.5	0.52	0.56	0.53	0.57	0.7	0.79	0.65
(6) Alcoholic cardiomyopathy	Patients (n)	74	92	131	121	134	158	174	177
	% in AUD	0.61	0.78	0.54	0.39	0.42	0.5	0.5	0.47
(7) Alcoholic gastritis	Patients (n)	195	190	463	519	584	574	544	523
	% in AUD	1.6	1.61	1.89	1.69	1.83	1.8	1.57	1.39
Any one of (3)–(7)	Patients (n)	2915	2963	5828	7952	8840	8014	8946	9189
	% in AUD	23.95	25.18	23.85	25.86	27.73	25.14	25.74	24.36
Any one of (1)–(2) without (3)–(7)	Patients (n)	9258	8806	18612	22607	21949	23210	25448	28255
	% in AUD	76.05	74.82	76.15	73.5	68.86	72.81	73.23	74.91
(8) Alcoholism counseling	Patients (n)	0	0	0	198	1087	652	357	277
	% in AUD	0	0	0	0.64	3.41	2.05	1.03	0.73
Anxiety/depressive disorders	Patients (n)	7213	7012	13935	18071	18334	18890	21116	23710
	% in AUD	59.25	59.58	57.02	58.76	57.52	59.26	60.76	62.86

**Table 2:** Descriptive statistics and results from logistic regression based on data from 2019.

	Descriptive statistics			Results from logistic regression		
	Patients with claims	Patients with AUD N(%)	p-value	Odds Ratio	95% CI	p-value
Overall	896695	37721 (4.2%)				
Gender: Female	530984	13858 (2.6%)	<0.001			
Male	365711	23863 (6.5%)		1.045	(1.044, 1.046)	<0.001
Age: 14 ≤ age < 18	94730	829 (0.9%)	<0.001			
18 ≤ Age ≤ 24	131925	2735 (2.1%)		1.005	(1.004, 1.007)	<0.001
25 ≤ Age ≤ 34	185121	8032 (4.3%)		1.017	(1.016, 1.019)	<0.001
35 ≤ Age ≤ 44	156650	8616 (5.5%)		1.023	(1.021, 1.024)	<0.001
45 ≤ Age ≤ 54	135894	8643 (6.4%)		1.028	(1.026, 1.030)	<0.001
55 ≤ Age ≤ 64	119469	7266 (6.1%)		1.026	(1.024, 1.027)	<0.001
65 ≤ Age	72906	1600 (2.2%)		1.001	(0.999, 1.003)	0.481
Race and Ethnicity:			<0.001			
Non-Hispanic White	635578	26776 (4.2%)				
Hispanic	18137	345 (1.9%)		0.995	(0.992, 0.998)	0.002
Non-Hispanic Black	90808	4432 (4.9%)		1.006	(1.005, 1.008)	<0.001
Non-Hispanic Missing	137060	5763 (4.2%)		0.999	(0.998, 1.000)	0.223
Non-Hispanic other	15112	405 (2.7%)		0.996	(0.993, 0.999)	0.013
RUC: Metro	428567	22942 (5.4%)	<0.001			
Non-metro	468128	14779 (3.2%)		0.982	(0.981, 0.983)	<0.001
Tobacco use: No	584819	10067 (1.7%)	<0.001			
Yes	311876	27654 (8.9%)		1.057	(1.056, 1.058)	<0.001
Alcoholism family history: No	895616	37193 (4.2%)	<0.001			
Yes	1079	528 (48.9%)		1.479	(1.462, 1.496)	<0.001
Anxiety/depression: No	570865	14011 (2.5%)	<0.001			
Yes	325830	23710 (7.3%)		1.042	(1.041, 1.043)	<0.001

**Table 3:** Number and percentage of patients with AUD received medication, rehabilitation and detoxification, and alcoholism counseling.

Year	2012	2013	2014	2015	2016	2017	2018	2019
Total patients with AUD (n)	12173	11769	24440	30756	31876	31876	34751	37721
Any medication below (n)	352	397	963	1694	2107	2357	2659	3066
%	2.89	3.37	3.94	5.51	6.61	7.39	7.65	8.13
Naltrexone (n)	50	62	220	507	756	1041	1262	1639
%	0.41	0.53	0.9	1.65	2.37	3.27	3.63	4.35
Disulfiram (n)	19	30	75	132	174	194	205	190
%	0.16	0.25	0.31	0.43	0.55	0.61	0.59	0.5
Acamprosate (n)	0	7	94	176	205	232	326	401
%	0	0.06	0.38	0.57	0.64	0.73	0.94	1.06
Topiramate (n)	287	305	624	986	1126	1080	1081	1105
%	2.36	2.59	2.55	3.21	3.53	3.39	3.11	2.93
Rehabilitation and detoxification (n)	0	0	15	553	769	843	1063	970
%	0	0	0.06	1.8	2.41	2.64	3.06	2.57
Counseling (n)	193	427	1383	2045	2938	3991	5607	7148
%	1.59	3.63	5.66	6.65	9.22	12.52	16.13	18.95
AUD counseling for 15 mins (n)	35	139	120	78	33	56	35	48
%	0.29	1.18	0.49	0.25	0.1	0.18	0.1	0.13
AUD counseling for 15–30 mins (n)	5	5	13	16	42	43	38	19
%	0.04	0.04	0.05	0.05	0.13	0.13	0.11	0.05
AUD counseling at least 3 hours/day and at least 3 days/week	19	9	222	431	1050	1921	3228	4389
%	0.16	0.08	0.91	1.4	3.29	6.03	9.29	11.64
Other AUD counseling&	134	275	1028	1520	1817	1975	2312	2698
%	1.1	2.34	4.21	4.94	5.7	6.2	6.65	7.15
Any treatment	539	792	2252	3924	4969	5966	7684	9307
%	4.43	6.73	9.21	12.76	15.59	18.72	22.11	24.67

**Note:** & Other AUD counseling refers to alcohol assessment, alcohol training service, alcohol group counseling, and alcohol hotline service.

**Table 4:** The utilization of treatments for AUD patients, classified by patients’ variables based on 2019 dataset.

		AUD patients (n)	Patients treated with medication	Patients treated with rehab and detox	Patients treated with counseling	Patients treated with any treatment
		37721	3066 (8.13%)	970 (2.57%)	7148 (18.95%)	9307 (24.67%)
Gender	Female	13858	1516 (10.9%)	329 (2.4%)	2817 (20.3%)	3 874 (28%)
	Male	23863	1550 (6.5%)	641 (2.7%)	4331 (18.1%)	5433 (22.8%)
Age	14 ≤ Age < 18	829	22 (2.7%)	1 (0.1%)	84 (10.1%)	104 (12.5%)
	18 ≤ Age ≤ 24	2735	154 (5.6%)	59 (2.2%)	603 (22%)	691 (25.3%)
	25 ≤ Age ≤ 34	8032	878 (10.9%)	375 (4.7%)	2302 (28.7%)	2875 (35.8%)
	35 ≤ Age ≤ 44	8616	940 (10.9%)	289 (3.4%)	2098 (24.4%)	2710 (31.5%)
	45 ≤ Age ≤ 54	8643	758 (8.8%)	178 (2.1%)	1337 (15.5%)	1944 (22.5%)
	55 ≤ Age ≤ 64	7266	314 (4.3%)	67 (0.9%)	671 (9.2%)	929 (12.8%)
	65 ≤ Age	1600	0 (0%)	1 (0.1%)	53 (3.3%)	54 (3.4%)
Race and Ethnicity	Hispanic	345	28 (8.1%)	5 (1.4%)	45 (13%)	66 (19.1%)
	Non-Hispanic black	4432	189 (4.3%)	86 (1.9%)	779 (17.6%)	917 (20.7%)
	Non-Hispanic missing	5763	376 (6.5%)	112 (1.9%)	940 (16.3%)	1228 (21.3%)
	Non-Hispanic other	405	28 (6.9%)	5 (1.2%)	80 (19.8%)	99 (24.4%)
	Non-Hispanic white	26776	2445 (9.1%)	762 (2.8%)	5304 (19.8%)	6997 (26.1%)
RUC	Metro	22942	1895 (8.3%)	760 (3.3%)	4813 (21%)	6116 (26.7%)
	Non-metro	14779	1171 (7.9%)	210 (1.4%)	2335 (15.8%)	3191 (21.6%)
Tobacco use	Yes	27654	2390 (8.6%)	856 (3.1%)	5416 (19.6%)	7042 (25.5%)
	No	10067	676 (6.7%)	114 (1.1%)	1732 (17.2%)	2265 (22.5%)
family history of Alcoholism	Yes	528	104(19.7%)	39(7.4%)	178 (33.7%)	230 (43.6%)
	No	37193	2962(8%)	931(2.5%)	6970 (18.7%)	9077 (24.4%)
Mental disorders	Yes	23710	2606(11%)	766(3.2%)	5125 (21.6%)	6903 (29.1%)
	No	14011	460(3.3%)	204(1.5%)	2023 (14.4%)	2404 (17.2%)

**Note:** Rural-Urban Continuum (RUC) codes between 1 and 3 indicates a metro area.

**Table 5:** The impact of variables on the utilization of treatments from logistic regression models in 2019.

	Medication		Rehab./detoxification		Counseling		Any treatment	
	OR	95% CI	OR	95% CI	OR	95% CI	OR	95% CI
Gender: Ref (Female)								
Male	0.971	(0.965,0.977)	1.01	(1.007,1.013)	1.007	(0.999,1.016)	0.986	(0.977,0.994)
Age: Ref (14 ≤ age < 18)								
18 ≤ Age ≤ 24	1.032	(1.010,1.054)	1.016	(1.004,1.029)	1.126	(1.093,1.16)	1.138	(1.102,1.175)
25 ≤ Age ≤ 34	1.086	(1.065,1.107)	1.039	(1.027,1.050)	1.194	(1.162,1.227)	1.255	(1.218,1.293)
35 ≤ Age ≤ 44	1.086	(1.065,1.107)	1.025	(1.013,1.036)	1.142	(1.111,1.174)	1.2	(1.165,1.236)
45 ≤ Age ≤ 54	1.068	(1.048,1.089)	1.011	(1.000,1.023)	1.046	(1.018,1.075)	1.102	(1.070,1.136)
55 ≤ Age ≤ 64	1.03	(1.011,1.051)	1.001	(0.989,1.012)	0.988	(0.961,1.016)	1.01	(0.981,1.041)
65 ≤ Age	0.989	(0.967,1.011)	0.995	(0.982,1.008)	0.937	(0.907,0.967)	0.926	(0.894,0.958)
Race and Ethnicity: Ref (Non-Hispanic White) Hispanic	1.008	(0.98,1.038)	0.986	(0.97,1.003)	0.92	(0.884,0.958)	0.929	(0.889,0.971)
Non-Hispanic Black	0.973	(0.965,0.982)	0.989	(0.984,0.994)	0.98	(0.968,0.993)	0.96	(0.947,0.974)
Non-Hispanic Missing	0.988	(0.981,0.996)	0.993	(0.988,0.997)	0.981	(0.971,0.992)	0.977	(0.965,0.988)
Non-Hispanic other	0.99	(0.965,1.017)	0.981	(0.966,0.996)	0.986	(0.95,1.023)	0.978	(0.939,1.019)
RUC: Ref (Metro)								
Non-metro	0.987	(0.98,0.995)	0.98	(0.977,0.984)	0.966	(0.956,0.977)	0.962	(0.951,0.973)
Tobacco use: Yes	1.006	(1.00,1.012)	1.016	(1.013,1.02)	1.016	(1.007,1.025)	1.013	(1.003,1.023)
Anxiety/depression: Yes	1.065	(1.059,1.071)	1.014	(1.011,1.018)	1.058	(1.049,1.066)	1.101	(1.091,1.11)
Alcoholism family history: Yes	1.089	(1.064,1.115)	1.039	(1.025,1.053)	1.12	(1.084,1.157)	1.148	(1.108,1.19)
