# Supplementary material for: Prevalence and Treatment for Alcohol Use Disorders Based on Kentucky Medicaid 2012–2019 Datasets
Source: J Alcohol Drug Depend. Author manuscript; Available in PMC 2023 Jan 19. (PMC9850928)
Supplement: Supplement to Manuscript [file NIHMS1837550-supplement-Supplement_to_Manuscript.pdf]

Supplementary Materials for the manuscript “Prevalence and treatment for alcohol use disorders based on Kentucky Medicaid 2012-2019 datasets” by Huirong Hu, Riten Mitra, Yuchen Han, Subhadip Pal, Haojing Huang, Vatsalya Vatsalya, Craig McClain, K.B. Kulasekera, and Maiying Kong

Table S1: ICD-9 and ICD-10 diagnosis codes for alcohol use disorders (AUD) and other comorbid variables.

**Alcohol use disorder**	ICD-9 diagnosis codes	ICD-10 diagnosis codes
(1) Nondependent abuse	305.0X	F10.1XX
(2) Alcohol dependence	303.XX	F10.2XX
(3) Alcohol associated liver diseases (ALD)		
Alcoholic fatty liver disease	571.0	K70.0
Acute hepatitis	571.1	K70.1, K7010, K7011
Alcoholic cirrhosis	571.2	K70.2, K70.3, K7030, K70.31
Alcoholic liver damage	571.3	K70.4, K70.40, K70.41, K709
(4) Alcohol induced mental disorder	291.X	F10.14, F10.15X, F10.18X, F10.24, F10.25X, F10.28X
(5) Alcoholic polyneuropathy	357.5	G62.1
(6) Alcoholic cardiomyopathy	425.5	I42.6
(7) Alcoholic gastritis	535.3X	K29.2X
**Comorbid variables**		
Tobacco use	305.1, V15.82	Z71.6, Z72.0, Z81.2, Z87.891, F17.X
Family history for alcoholism	V61.41	Z81.1
Mental disorders		
Anxiety	293.84, 300.XX, 309.81	F40.XXX, F41.XXX, F42.XXX, F44.9, F45.8, F48.8, F48.9, F99, R45.2, F45.5, F45.6, F45.7, F06.4
Major depressive disorder (MDD)	296.2X, 296.3X, 300.4, 311	F32.XX, F33.XX, F34.1

Table S2: ICD-9 procedure codes and HCPCS procedure codes for alcohol rehabilitation/detoxification and alcoholism counseling.

Behavior Therapy	ICD-9 Procedure Codes	HCPCS Procedure Codes
Alcohol Rehabilitation & Detoxification	00946, 09461, 09462, 09463, 09467, 09468, 09469	H0008, H0009, H0010, H0011, H0012, H0013, H0014
Alcoholism counseling	V65.42, Z71.41	G0443, G0396, G0397, G0443, H0001, H0004, H0005, H0050, H0015, H0021, H0030
Counseling interval 15 Min		H0004, H0050
Counseling interval 15 - 30 Min		G0396
AUD counseling at least 3 hours/day and at least 3 days/week		H0015
Other AUD counseling [&]		H0001, H0005, H0021, H0030
Alcohol misuse counseling		G0443

Note: & Other AUD counseling includes alcohol assessment, alcohol training service, alcohol group counseling, and alcohol hotline service

Table S3: Drug codes for drugs which could be used to treat patients with AUD.

Drug	Drug codes
Naltrexone Hydrochloride	00406117001, 00406117003, 16729008101, 16729008110, 47335032683, 47335032688, 51224020630, 51224020650, 68084029121, 68094085362, 68084029111, 00555090201, 00555090202, 00185003901, 51927437700, 38779088703, 38779088704
Acamprosate Calcium	00093535286, 00378633380, 60687012125, 68382056928, 68462043518, 00258400060, 51079024106
Disulfiram	00054035613, 00054035625, 00054035713, 00054035725, 00093503501, 00093503601, 47781060730, 64980017103, 64980017203, 64980017101, 64980071202, 00378414001, 00378414101, 00603343221
Topiramate	00093015506, 00093015510, 00093721906, 00093721910, 00093722006, 00093722010, 00093733506, 00093733606, 00093754006, 00093754010, 00378610105, 00378610191, 00378610205, 00378610291, 00378610305, 00378610391, 00378610591, 00781227660, 13668003105, 13668003160, 13668003205, 13668003260, 13668003305, 13668003360, 13668003405, 13668003460, 16252056860, 16252056960, 31722027805, 31722027810, 31722027860, 31722027905, 31722027910, 31722027960, 31722028005, 31722028010, 31722028060, 31722028105, 31722028110, 31722028160, 51079072620, 51079072720, 51079072820, 59762103001, 59762103101, 59762103201, 59762103301, 60429077010, 60505276006, 60505276106, 60505276206, 60505276306, 62756070713, 62756070786, 62756071013, 62756071086, 62756071113, 62756071186, 62756071213, 62756071286, 64376012101, 64376012110, 68084034211, 68084034401, 68084034411, 68084034521, 68382000414, 68382000514, 68382013805, 68382013814, 68382013905, 68382013914, 68382014005, 68382014014, 68382014105, 68382014114, 68462010810, 68462010860, 68462010910, 68462010960, 68462011010, 68462011060, 68462015310, 68462015360, 60505276005, 60505276105, 65862017360, 65862017460, 00832107130, 00832107430, 00832107530, 60505276205, 60505276305, 68084034201, 00832107230, 00832107315, 00832107330, 00832107415, 29300011710, 38779244308, 47335070713, 47335070786, 47335071013, 47335071086, 47335071113, 47335071186, 47335071213, 47335071286, 51079072601, 51927467100, 69097012203, 69097012212, 69097012215, 69097012303, 69097012312, 69097012315, 69097012403, 69097012412, 69097012415, 69097012503, 69097012512, 69097012515, 29300011610, 51552120605, 69097081603, 69097081615, 69097081703, 69097081715, 69097081803, 69097081815, 69097081903, 29300011505, 29300011510, 29300011605, 29300011616, 29300011705, 29300011805, 29300011816, 29300011516, 29300011716, 00395815156, 29300011810, 38779244305, 69097081915

Table S4: Summary for the number of Medicaid patients by demographics and geographics in each year.

	Year	2012	2013	2014	2015	2016	2017	2018	2019
Number of patients		471415	463989	791575	891448	916002	919921	915672	896695
Gender	female	308909(65.5%)	303105(65.3%)	476052(60.1%)	527052(59.1%)	540564(59.0%)	542920(59.0%)	540712(59.1%)	530984(59.2%)
	Male	162506(34.5%)	160884(34.7%)	315523(39.9%)	364396(40.9%)	375438(41.0%)	377001(41.0%)	374960(41.0%)	365711(40.8%)
Age	[14, 18]	77780(16.5%)	77387(16.7%)	83310(10.5%)	87663(9.8%)	91234(9.96%)	93489(10.2%)	95594(10.4%)	94730(10.6%)
	[18, 24]	67419(14.3%)	64470(13.9%)	117591(14.9%)	135307(15.2%)	137777(15.0%)	136905(14.9%)	136176(14.9%)	131925(14.7%)
	[25, 34]	78649(16.7%)	76179(16.4%)	163437(20.7%)	189615(21.3%)	193988(21.2%)	194059(21.1%)	191567(20.9%)	185121(20.6%)
	[35, 44]	59176(12.6%)	57747(12.5%)	131626(16.6%)	153466(17.2%)	158695(17.3%)	159179(17.3%)	159615(17.4%)	156650(17.5%)
	[45, 54]	63823(13.5%)	62139(13.4%)	128550(16.2%)	144249(16.2%)	146494(16.0%)	145491(15.8%)	141597(15.5%)	135894(15.2%)
	[55, 65]	53140(11.3%)	54681(11.8%)	95621(12.1%)	109488(12.3%)	115414(12.6%)	118346(12.9%)	118791(13.0%)	119469(13.3%)
	> 65	71428(15.2%)	71386(15.4%)	71440(9.0%)	71660(8.0%)	72400(7.9%)	72452(7.9%)	72332(7.9%)	72906(8.13%)
Metro	Metro	200340(42.5%)	196944(42.5%)	357996(45.2%)	414198(46.5%)	428560(46.8%)	431937(47.0%)	436079(47.6%)	428567(47.8%)
	Non-metro	271071(57.5%)	267043(57.6%)	433563(54.8%)	477246(53.5%)	487440(53.2%)	487981(53.1%)	479591(52.4%)	468128(52.2%)
Medical Regions	MED Region 1	23167(4.91%)	22587(4.87%)	38827(4.9%)	43980(4.93%)	45469(5.0%)	46373(5.0%)	46655(5.1%)	44996(5.02%)
	MED Region 2	40327(8.55%)	39491(8.5%)	64770(8.2%)	74803(8.39%)	77582(8.5%)	78196(8.5%)	78199(8.5%)	76197(8.5%)
	MED Region 3	100581(21.3%)	98949(21.3%)	182296(23.0%)	211099(23.7%)	217438(23.7%)	218796(23.8%)	218670(23.9%)	214289(23.9%)
	MED Region 4	65678(13.9%)	64492(13.9%)	107248(13.6%)	119273(13.4%)	121567(13.3%)	122287(13.3%)	121684(13.3%)	121046(13.5%)
	MED Region 5	76336(16.2%)	75708(16.3%)	136829(17.3%)	155750(17.5%)	160716(17.6%)	161589(17.6%)	162040(17.7%)	159618(17.8%)
	MED Region 6	27521(5.84%)	27054(5.8%)	50125(6.33%)	57816(6.49%)	59583(6.5%)	59903(6.51%)	59479(6.5%)	58246(6.5%)
	MED Region 7	35687(7.57%)	34926(7.53%)	57356(7.25%)	62971(7.06%)	64567(7.05%)	65127(7.1%)	64696(7.07%)	62514(6.97%)
	MED Region 8	102114(21.66%)	100780(21.7%)	154108(19.47%)	165752(18.6%)	169078(18.5%)	167647(18.2%)	164247(17.9%)	159789(17.8%)
Race & Ethnicity	Non-Hispanic White	327380(69.4%)	318554(68.7%)	535904(67.7%)	611248(68.6%)	644632(70.4%)	655816(71.3%)	652449(71.3%)	635578(70.9%)
	Non-Hispanic Black	43072(9.1%)	42143(9.1%)	71225(9%)	82309(9.2%)	87911(9.6%)	90572(9.8%)	92191(10.1%)	90808(10.1%)
	Non-Hispanic Other	11140(2.4%)	11628(2.5%)	46473(5.9%)	31299(3.5%)	13862(1.5%)	14205(1.5%)	14807(1.6%)	15112(1.7%)
	Non-Hispanic missing	84233(17.9%)	85816(18.5%)	128569(16.2%)	154500(17.3%)	154454(16.9%)	142609(15.5%)	138740(15.2%)	137060(15.3%)
	Hispanic	5590(1.2%)	5848(1.3%)	9404(1.2%)	12092(1.4%)	15143(1.7%)	16719(1.8%)	17485(1.9%)	18137(2%)

Note: Each year refers from January 1 to December 31 of that year.

Table S5: Descriptive statistics summarized as sample sizes and AUD prevalence rates, stratified by patients' variables.

Year	2012		2013		2014		2015		2016		2017		2018	
Patients	471415	12173 (2.6%)	463989	11769 (2.5%)	791575	24440 (3.1%)	891448	30756 (3.5%)	916002	31876 (3.5%)	919921	31876 (3.5%)	915672	34751 (3.8%)
**Sex:** Female	308909	4665 (1.5%)	303105	4505 (1.5%)	476052	8604 (1.8%)	527052	10870 (2.1%)	540564	11160 (2.1%)	542920	10998 (2%)	540712	12527 (2.3%)
Male	162506	7508 (4.6%)	160884	7264 (4.5%)	315523	15836(5%)	364396	19886 (5.5%)	375438	20716 (5.5%)	377001	20878 (5.5%)	374960	22224 (5.9%)
**Age:**														
14 ≤ Age < 18	77780	817 (1.1%)	77387	739 (1%)	83310	720 (0.9%)	87663	929 (1.1%)	91234	1140 (1.2%)	93489	894 (1%)	95594	864 (0.9%)
18 ≤ Age ≤ 24	67419	696 (1%)	64470	604 (0.9%)	117591	1683 (1.4%)	135307	2246(1.7%)	137777	2318(1.7%)	136905	2229(1.6%)	136176	2513(1.8%)
25 ≤ Age ≤ 34	78649	1634 (2.1%)	76179	1487 (2%)	163437	4288 (2.6%)	189615	5813 (3.1%)	193988	6083 (3.1%)	194059	6299 (3.2%)	191567	7019 (3.7%)
35 ≤ Age ≤ 44	59176	2106 (3.6%)	57747	2043 (3.5%)	131626	5267 (4%)	153466	6644 (4.3%)	158695	7053 (4.4%)	159179	6967 (4.4%)	159615	7790 (4.9%)
45 ≤ Age ≤ 54	63823	3521 (5.5%)	62139	3435 (5.5%)	128550	7263 (5.6%)	144249	8734 (6.1%)	146494	8430 (5.8%)	145491	8260 (5.7%)	141597	8455 (6%)
55 ≤ Age ≤ 64	53140	2394 (4.5%)	54681	2498 (4.6%)	95621	4224 (4.4%)	109488	5255 (4.8%)	115414	5632 (4.9%)	118346	5950 (5%)	118791	6714 (5.7%)
65 ≤ Age	71428	1005 (1.4%)	71386	963 (1.3%)	71440	995 (1.4%)	71660	1135 (1.6%)	72400	1220 (1.7%)	72452	1277 (1.8%)	72332	1396 (1.9%)
**Race &Ethnicity:**														
Hispanic	5590	50 (0.9%)	5848	62 (1.1%)	9404	155 (1.6%)	12092	232 (1.9%)	15143	275 (1.8%)	16719	269 (1.6%)	17485	341 (2%)
Non-Hispanic Black	43072	1248 (2.9%)	42143	1172 (2.8%)	71225	2469 (3.5%)	82309	3162 (3.8%)	87911	3221 (3.7%)	90572	3419 (3.8%)	92191	4021 (4.4%)
Non-Hispanic missing	84233	2533 (3%)	85816	2602 (3%)	128569	4382 (3.4%)	154500	5737 (3.7%)	154454	5688 (3.7%)	142609	4900 (3.4%)	138740	5097 (3.7%)
Non-Hispanic Other	11140	172 (1.5%)	11628	202 (1.7%)	46473	1503 (3.2%)	31299	653 (2.1%)	13862	276 (2%)	14205	262 (1.8%)	14807	362 (2.4%)
Non-Hispanic White	327380	8170 (2.5%)	318554	7731 (2.4%)	535904	15931(3%)	611248	20972 (3.4%)	644632	22416 (3.5%)	655816	23026 (3.5%)	652449	24930 (3.8%)
**RUC:** Metro	200340	5983 (3%)	196944	5829(3%)	357996	13581(3.8%)	414198	17412 (4.2%)	428560	18026 (4.2%)	431937	18438 (4.3%)	436079	20986 (4.8%)
Non-metro	271071	6190 (2.3%)	267043	5940(2.2%)	433563	10859(2.5%)	477246	13344 (2.8%)	487440	13850 (2.8%)	487981	13438 (2.8%)	479591	13765 (2.9%)
**Tobacco Use**:														
No	365699	3975 (1.1%)	356949	3752 (1.1%)	573425	7482 (1.3%)	622847	9360 (1.5%)	614864	9272 (1.5%)	605561	8765 (1.4%)	601297	9600 (1.6%)
Yes	105716	8198 (7.8%)	107040	8017 (7.5%)	218150	16958(7.8%)	268601	21396 (8%)	301138	22604 (7.5%)	314360	23111 (7.4%)	314375	25151 (8%)
**Family history of alcoholism:**														
No	471322	12146 (2.6%)	463952	11756 (2.5%)	791458	24378(3.1%)	891174	30633 (3.4%)	915256	31569 (3.4%)	918946	31500 (3.4%)	914616	34263 (3.7%)
Yes	93	27 (29%)	37	13 (35.1%)	117	62 (53%)	274	123 (44.9%)	746	307 (41.2%)	975	376 (38.6%)	1056	488 (46.2%)
Mental Disorders: No	326048	4960 (1.5%)	321278	4757 (1.5%)	555555	10505(1.9%)	612764	12685 (2.1%)	619713	13542 (2.2%)	609903	12986 (2.1%)	599078	13635 (2.3%)
Yes	145367	7213 (5%)	142711	7012 (4.9%)	236020	13935(5.9%)	278684	18071 (6.5%)	296289	18334 (6.2%)	310018	18890 (6.1%)	316594	21116 (6.7%)

Note: Rural-Urban Continuum (RUC) codes between 1 and 3 indicates a metro area.
